# Low Risk Monitoring in Neurocritical Care

**DOI:** 10.3389/fneur.2018.00938

**Published:** 2018-11-06

**Authors:** Christian D. Becker, Christian Bowers, Dipak Chandy, Chad Cole, Meic H. Schmidt, Corey Scurlock

**Affiliations:** ^1^eHealth Center, Westchester Medical Center Health Network, Valhalla, NY, United States; ^2^Department of Medicine, New York Medical College, Valhalla, NY, United States; ^3^Division of Pulmonary and Critical Care Medicine, Department of Medicine, Westchester Medical Center, Valhalla, NY, United States; ^4^Department of Neurosurgery, New York Medical College, Valhalla, NY, United States; ^5^Department of Neurosurgery, Westchester Medical Center, Valhalla, NY, United States; ^6^Department of Anesthesiology, Westchester Medical Center, Valhalla, NY, United States

**Keywords:** low risk monitor, telemedicine, tele-ICU, electronic ICU, neuroscience ICU, neuro-ICU

## Abstract

**Background/Rationale:** Patients are admitted to Intensive care units (ICUs) either because they need close monitoring despite a low risk of hospital mortality (LRM group) or to receive ICU specific active treatments (AT group). The characteristics and differential outcomes of LRM patients vs. AT patients in Neurocritical Care Units are poorly understood.

**Methods:** We classified 1,702 patients admitted to our tertiary and quaternary care center Neuroscience-ICU in 2016 and 2017 into LRM vs. AT groups. We compared demographics, admission diagnosis, goal of care status, readmission rates and managing attending specialty extracted from the medical record between groups. Acute Physiology, Age and Chronic Health Evaluation (APACHE) IVa risk predictive modeling was used to assess comparative risks for ICU and hospital mortality and length of stay between groups.

**Results:** 56.9% of patients admitted to our Neuroscience-ICU in 2016 and 2017 were classified as LRM, whereas 43.1% of patients were classified as AT. While demographically similar, the groups differed significantly in all risk predictive outcome measures [APACHE IVa scores, actual and predicted ICU and hospital mortality (*p* < 0.0001 for all metrics)]. The most common admitting diagnosis overall, cerebrovascular accident/stroke, was represented in the LRM and AT groups with similar frequency [24.3 vs. 21.3%, respectively (*p* = 0.15)], illustrating that further differentiating factors like symptom duration, neurologic status and its dynamic changes and neuro-imaging characteristics determine the indication for active treatment vs. observation. Patients with intracranial hemorrhage/hematoma were significantly more likely to receive active treatments as opposed to having a primary focus on monitoring [13.6 vs. 9.8%, respectively (*p* = 0.017)].

**Conclusion:** The majority of patients admitted to our Neuroscience ICU (56.9%) had <10% hospital mortality risk and a focus on monitoring, whereas the remaining 43.1% of patients received active treatments in their first ICU day. LRM Patients exhibited significantly lower APACHE IVa scores, ICU and hospital mortality rates compared to AT patients. Observed-over-expected ICU and hospital mortality ratios were better than predicted by APACHE IVa for low risk monitored patients and close to prediction for actively treated patients, suggesting that at least a subset of LRM patients may safely and more cost effectively be cared for in intermediate level care settings.

## Introduction

General guidelines for intensive care unit (ICU) admission, discharge and triage have been developed by the Society of Critical Care Medicine in 1999 and updated in 2016 (1). They provide an important framework to guide triage and ICU bed utilization, but inevitably represent only general guidelines. Conceptually, all ICU bed utilization approaches attempt to factor in acuity of the patient's illness at presentation as well as changes of acuity over time combined with complexity of management. ICU patient populations can also dichotomously be divided into those who receive active ICU treatments for impaired organ functions and those who are not, but are being monitored because they are at high risk of impaired or worsening organ function despite an overall low hospital mortality risk. Given that ICU beds are a precious resource in hospitals and societal forces will place pressures on health care spending, understanding optimal ICU utilization will be paramount in the next two decades (2).

### APACHE

A multitude of tools have been developed for ICU performance assessments (3) and comprehensive systematic performance measurements (4). *Acute Physiology, Age and Chronic Health Evaluation* (APACHE) scoring was developed in the late 1970s as a tool to describe groups of patients based on severity of illness and enable risk prediction modeling for ICU and hospital mortality and length of stay (5). Over four consecutive iterations APACHE [APACHE in 1981 (5), APACHE II in 1985 (6), APACHE III in 1991 (7) and APACHE IV in 2006 (8)] has evolved into and emerged as the gold standard risk prediction tool for critically ill patients. There are a variety of well-validated disease or condition specific risk prediction tools in Neurocritical Care, among them the Hunt&Hess (HH) and World Federation of Neurological Surgeons (WFNS) grading systems for Subarachnoid Hemorrhage (SAH), the intracerebral hemorrhage (ICH) score or the ischemic Stroke Prediction Risk Score (iScore). These risk prediction tools were developed and validated to guide clinical care and generate comparative risk predictions for patients with specific diagnoses. In other words, they generate comparative risk predictions ***within diagnostic categories*** but fall short when it comes to generating comparative predictions ***across the spectrum of critical illness*** with a unifying validated methodology.

### Active treatment vs. monitoring

In parallel to capturing the acuity of ICU patients, the Therapeutic Intervention Scoring System (TISS) was developed in 1974 (9) and updated in 1983 (10) to classify critically ill patients by therapeutic interventions. The TISS nomenclature categorizes patients receiving one or more of 35 active ICU treatments (AT) *as specified by the TISS* during their first 24 h in the ICU are “actively treated,” whereas those who do not receive any active treatments in their first ICU day are categorized as “monitored.” The same group that developed APACHE published on further predictive multivariate equations in 1995 that aim to estimate the probability of need of life-supporting treatments as defined by TISS (11). Factors found to be most important to determine this probability were: diagnosis, APACHE III score, age, operative status and prior location in hospital as well as duration of hospitalization (11, 12). Taking this a step further, a model was developed to predict the likelihood of low risk monitor patients receiving one of the same 35 active life-supporting treatments as defined by TISS *after* their first ICU day in an attempt to identify patients who may not need ICU admission (13).

### Low risk monitoring

The composite metric of Low Risk Monitoring (LRM) has been developed based on APACHE and TISS to assess ICU resource utilization, specifically determining the percentage of ICU patients admitted to the ICU for monitoring rather than receiving treatment(s) that by general consensus typically require an ICU setting. LRM is defined as the fraction of ICU patients with <10% risk of ICU mortality as determined by APACHE scoring (“*low risk”* component) who do not get ICU specific treatments in their first ICU day or “APACHE Day” as defined by APACHE rules (“*monitoring”* component). The rationale for knowing the percentage of ICU patients who receive monitoring but no active treatments during their first ICU day is that a fraction of these patients may not require precious ICU resources and therefore could more cost-effectively be managed in a lower level care setting.

For the purpose of calculating LRM%, “active treatment” is defined not by subjective assessment, but by TISS. This is important as it illustrates that there are potentially subgroups of patients who are receiving treatments different from the 35 TISS treatments. These patients would be classified as “monitored” by TISS, even though their non-TISS treatment may be labor-intensive and necessitate and benefit from an ICU setting. Having said that, the TISS list was designed to be inclusive and capture ideally all relevant ICU related treatments. It was, however, last updated over 34 years ago. It is unclear to what extent TISS is missing phenotypes of ICU patients in different specialty ICUs and whether those patients are best cared for in an ICU setting. On the other hand, it is likely that potential novel non-TISS ICU treatments will be indirectly captured, because they necessitate other TISS ICU treatments along with them (i.e., patients undergoing invasive CNS monitoring techniques will also need to be sedated and mechanically ventilated). A very important aspect of triage decisions and ICU utilization are nurse-to-patient ratios. There is data to suggest that a portion of LRM ICU admissions might be driven by patients with rather uncomplicated monitoring requirements but primarily intensive nursing needs (14). These patients too, would benefit from the ICU setting.

A commonly used framework for monitoring LRM as an ICU utilization metric is based on tele-ICU monitoring technology and software used in ~11–15% of ICUs in the US (eCare Manager™, Philips, Netherlands) (15, 16). There are some notable differences between the TISS active ICU specific treatments and APACHE active treatments as used by the tele-ICU monitoring software we are reporting on which are summarized in Table 1.

**Table 1:** List of “active treatments” by organ system (left column) and compared as per therapeutic intervention scoring system (TISS) (second column) and Acute Physiology, Age and Chronic Health Evaluation (APACHE) Iva/tele-intensive care unit monitoring software (third column).

	**Active treatments per**		
**Category**	**TISS 9, 10**	**APACHE 5–8**	**Substantive Difference**
Respiratory	Controlled ventilation	Controlled ventilation	None
	Controlled ventilation with muscle relaxant	Controlled ventilation with muscle relaxant	None
	IMV or Assisted Ventilation	IMV or Assisted Respirations	None
	Spontaneous PEEP or CPAP	Spontaneous PEEP or CPAP or BiPAP	BiPAP included in APACHE
	Nasal or oral intubation	Nasal or oral intubation (ICU)	Qualifier “Emergency” in TISS; Qualifier “ICU” in APACHE
	Fresh tracheostomy (<48 h)	Fresh tracheostomy (<48 h)	None
	Emergency bronchoscopy	Bronchoscopy (ICU)	Qualifier “Emergency” in TISS; Qualifier “ICU” in APACHE
Cardiovascular	Atrial or ventricular pacing	Atrial or Ventricular pacing (active)	Qualifier “Active” included in APACHE
	Intraaortic balloon	IABP (active)	None
	Vasoactive drug (1)	Vasoactive drug (1)	None
	Vasoactive drugs (>1)	Vasoactive drugs (>1)	None
	Intravenous antiarrhythmic	Continuous antiarrhythmic IV	Qualifier “Continuous” included in APACHE
	>6 L/d intravenous fluids	>6 L/d intravenous fluids	None
	Rapid blood transfusion	Rapid blood transfusion	None
	Post-arrest	Post-arrest (24 h)	Qualifier “24 h” added in APACHE
	Trauma suit	Trauma suit	None
	Cardioversion	Cardioversion	None
	Pericardiocentesis	Pericardiocentesis (ICU)	Qualifier “Emergency” in TISS; Qualifier “ICU” in APACHE
Renal	Stable hemodialysis	Stable hemodialysis	None
	Unstable hemodialysis	Unstable hemodialysis	None
Gastrointestinal	Intravenous pitressin infusion	Intravenous vasopressin infusion	None
	Continuous arterial drug infusion	Continuous arterial drug infusion	None
	Balloon tamponade for esophageal varices	Balloon tamponade for esophageal varices	None
	Continuous nasogastric lavage	Continuous nasogastric lavage	None
	Emergency endoscopy	Endoscopy (ICU)	Qualifier “Emergency” in TISS; Qualifier “ICU” in APACHE
Neurologic	Mannitol infusion	Mannitol infusion (continuous or intermittent)	Qualifier “continuous or intermittent” added in APACHE
	Ventriculostomy	Ventriculostomy	None
	Treatment of Seizure	Seizure (active)/treatment of metabolic encephalopathy	Treatment of metabolic encephalopathy added in APACHE
	Induced hypothermia	Induced hypothermia (<32C)	None
	Barbiturate anesthesia	Barbiturate anesthesia	None
Miscellaneous	Treatment of metabolic acidosis or alkalosis	Treatment of acidosis/alkalosis (complex)	None
	Emergency operation	Emergency operative procedure	None
	Concentrated K intravenously	n/a	Not included In APACHE
	Complex metabolic balance	n/a	Not included In APACHE
	Active diuresis for fluid overload	n/a	Not included In APACHE

*The substantive differences between the two frameworks are summarized in the right column*.

The characteristics and differential outcomes of LRM patients in Neuroscience Critical Care Units are sparsely reported on. The purpose of this study is to describe the LRM patient population and compare it to actively treated patients (AT) in our tertiary/quaternary care center Neuroscience-ICU. We also discuss the practice guidelines, policies, and expert consensus recommendations that address and influence LRM triage decisions for the Neuroscience ICU patient population.

## Materials and methods

The Institutional Review Board of New York Medical College/Westchester Medical Center approved this study under “exempt approval” category (Reference number WMC L12-240). Patients admitted to the Westchester Medical Center Neuroscience-ICU in 2016 and 2017 were retrospectively identified. The following patients were excluded from the analyses: patients with ICU length of stay (LOS) < 4 h, patients younger than 18 years of age, patients with invalid data points and patients with missing data for APACHE IVa risk predictive modeling. Patients were categorized according to whether they received one or more of 32 ICU specific treatments in their first ICU day (AT, Active Treatment) or not (LRM, Low Risk Monitoring). We used APACHE IVa risk predictive modeling built into our existing eICU coverage model (eCare Manager 4.0.1, Royal Philips, Netherlands) to assess AT vs. LRM comparative risks for ICU and hospital mortality and length of stay. All patient characteristics like admission diagnosis, primary managing subspecialty as well as data for risk predictive modeling were extracted from the deidentified eCare Manager derived research database (eSearch 5.2; Royal Philips, Netherlands). Admission diagnosis categories follow APACHE IVa methodology. Every admission to the Neuroscience ICU was reviewed in detail and in real time by a board certified intensivist, who then entered admission source and comorbidity information necessary for APACHE IVa predictive scoring and assigned the most appropriate out of APACHE IVa non-surgical and surgical Neuroscience diagnostic categories (30 medical admission diagnostic categories: neurologic abscess, amyotrophic lateral sclerosis, coma, cerebrovascular accident/stroke, drug withdrawal, encephalitis, encephalopathies, Guillain-Barre syndrome, epidural hematoma, subdural hematoma, intracranial hemorrhage/hematoma, obstructive hydrocephalus, meningitis, myasthenia gravis, neurologic neoplasm, non-traumatic coma due to anoxia/ischemia, nine categories of Overdoses, cranial nerve palsy, carbon monoxide/arsenic/cyanide poisoning, seizures, subarachnoid hemorrhage and Neurologic Medical, Other; 26 surgical admission diagnostic categories: surgery for abscess/infection, vascular anastomosis, surgery for arteriovenous malformation, brain biopsy, Burr hole placement, surgery for cerebrospinal fluid leak, surgery for complications of previous spinal cord surgery, cranial nerve decompression/ligation, cranioplasty and complications from previous craniotomies, devices for spine fracture/dislocation, spinal fusion, surgery for subdural hematoma, surgery for epidural hematoma, surgery for intracranial hemorrhage/hematoma, laminectomy, surgery for cranial neoplasm, surgery for spinal cord neoplasm, surgery for intractable seizures, shunts and revisions, other spinal cord surgery, stereotactic procedure, surgery for subarachnoid hemorrhage/intracranial aneurysm, sympathectomy, transsphenoidal surgery, ventriculostomy and Neurologic Surgery, Other). For both surgical and medical admissions the categories Neurologic Surgery, Other or Neurologic Medical, Other were selected if no other available category provided a more accurate classification. Univariate statistical comparisons of patient characteristics were performed using the unpaired *t*-test for continuous variables and Fisher's exact test for dichotomous variables, both with a significance criterion of *p* < 0.05 (Graphpad Prism, Graphpad Software, US).

## Results

There were a total of 1,724 admissions to the Neuroscience-ICU in 2016 and 2017. Seventeen admissions were excluded due to ICU LOS < 4 h, four admissions were excluded due to missing Admit source and one admission was excluded due to missing hospital discharge location. 1,702 (98.7%) admissions without any exclusion criteria remained for 2016 and 2017 and were included in the analysis. 968 (56.9%) of those 1,702 admissions were in the LRM category, and 734 (43.1%) were in the AT group. The mean age was not statistically different between the LRM and AT groups (61.2 vs. 62.8 years, *p* = 0.83). 40.1% of patients in the LRM group and 39.5% of patients in the AT group were female. The ethnicity distribution was very similar in both groups (see Table 2). A similar percentage of patients opted for “Do not resuscitate” status in the LRM and AT groups (9.1 vs. 8.9%, respectively). Readmission rates were similar in the LRM and AT groups, both within 48 h of ICU discharge and later than 48 h after ICU discharge (LRM group: 1.4% < 48 h, 5.4% overall, AT group: 2.5% < 48 h, 4.8% overall). Patients in the AT group had significantly higher APACHE IVa scores compared to the LRM group (57.3 ± 0.91 vs. 39.8 ± 0.49, *p* < 0.0001). The top 7 admission diagnoses overall were cerebrovascular accident/stroke (391 cases, 23.0%), intracranial hemorrhage/hematoma (195 cases, 11.5%), other neurologic surgery (79 cases, 4.6%), surgery for cranial neoplasms (78 cases, 4.6%), surgery for subarachnoid hemorrhage (75 cases, 4.4%), medically managed subarachnoid hemorrhage (72 cases, 4.2%) and Seizures (64 cases, 3.8%). The top seven admission diagnoses overall accounted for 54.4% of LRM admissions and 58.2% of AT admissions.

**Table 2:** Baseline patient characteristics and outcomes of low risk monitoring compared to active treatment admissions.

**Neuroscience-ICU 2016–2017**	**LRM**	**AT**	***p*-value**
No. of ICU admissions (%)	968 (56.9)	734 (43.1)
Age (mean ± SEM)	61.2 ± 0.58	62.8 ± 0.63	0.06 (NS)
Female %	40.1	39.5	0.83 (NS)
Caucasian %	67.9	71.3	0.14 (NS)
African American %	10.7	10.1	0.69 (NS)
Hispanic %	8.9	9.4	0.73 (NS)
Asian %	2.2	1.2	0.19 (NS)
Other %	10.3	8.0	0.12 (NS)
Do not resuscitate status %	9.1	8.9	0.93 (NS)
Readmission %	5.4	4.8	0.66 (NS)
Readmission < 48 h %	1.4	2.5	0.15 (NS)
APACHE IVa (mean ± SEM)	39.8 ± 0.49	57.3 ± 0.91	<0.0001 (^****^)
Top 7 admission diagnoses (*n*, %)	527 (54.4)	427 (58.2)	0.13 (NS)
Cerebrovascular accident/Stroke	235 (24.3)	156 (21.3)	0.15 (NS)
Intracranial hemorrhage	95 (9.8)	100 (13.6)	0.017 (^*^)
Neurologic surgery, Other	48 (5.0)	31 (4.2)	0.49 (NS)
Surgery for cranial neoplasms	39 (4.0)	39 (5.3)	0.24 (NS)
Surgery for subarachnoid hemorrhage	54 (5.6)	21 (2.9)	0.008 (^**^)
Subarachnoid hemorrhage	28 (2.9)	44 (6.0)	0.002 (^***^)
Seizures	28 (2.9)	36 (4.9)	0.039 (^*^)
Actual ICU mortality (*n*, %)	8 (0.8)	113 (15.4)	<0.00001 (^*****^)
Predicted ICU mortality (%)	3.2	14	<0.0001 (^****^)
Actual Hospital mortality *n*, (%)	36 (3.7)	162 (22.1)	<0.0001 (^****^)
Predicted hospital mortality (%)	7.4	21.9	<0.0001 (^****^)
Primary neurosurgery Mgmt (%)	67.3	68.9	0.49 (NS)
Primary neurology Mgmt (%)	15.9	13.4	0.17 (NS)
Primary internal medicine/CCM management (%)	2.1	5.2	0.0003 (^***^)
Primary Mgmt by other (%)	14.7	12.5	0.15 (NS)

APACHE IVa scores are measured as dimensionless whole numbers between 0 and 200. NS, non-significant. Asterisks denote increasing levels of statistical significance:

* *p < 0.05*,

** *p < 0.01*,

*** *p < 0.001*,

**** *p < 0.0001*,

***** *p < 0.00001*.

Actual ICU mortality was 0.8% in the LRM group vs. 15.4% in the AT group. Actual hospital mortality was 3.7% in the LRM group vs. 22.1% in the AT group. The ratios of observed-to-predicted ICU and hospital mortality were below prediction for the LRM group (0.25 and 0.50, respectively). The ratios of observed-to-predicted ICU and hospital mortality were at or slightly above prediction for the AT group (1.1 and 1.0, respectively).

Neurosurgery primarily managed 67.3% of LRM patients and 68.9% of AT group patients, whereas Neurology managed 15.9% of LRM patients and 13.4% of AT patients. There was a significantly higher admixture of medically critically ill patients primarily managed by Pulmonary/Critical Care Medicine in the AT group vs. the LRM group [5.2 vs. 2.1%, respectively (*p* = 0.0003)]. All patient characteristics (average APACHE scores, predicted ICU and hospital mortality, actual and predicted ICU and hospital length of stay) were statistically significantly different between LRM and AT patients (see Table 2).

## Discussion

A reasonable and universally accepted standard of LRM% for ICUs in general and for different subspecialty vs. mixed patient populations does not exist. In general, a national average across *all comers* ICUs in the United States (US) today of LRM% in ICU settings is around 35.4% of low risk patient stays (14). A variety of factors influence observed LRM% between different patient populations, ICUs and hospitals (also see Table 3):

1. Hospital geographic setting (urban vs. rural),
2. Hospital overall size as well as ICU bed to general floor bed ratios,
3. Hospital category (academic vs. community vs. hybrid),
4. Presence or absence of intermediary or specialty care units,
5. Staffing models (low vs. high intensity staffing for MDs, RNs and other ancillary staff),
6. Overall Hospital ICU bed utilization/occupancy rates.

**Table 3:** List of factors influencing the percentage of low risk monitoring patients for any given hospital and intensive care unit (ICU).

Hospital	Geographic setting	
		Urban
		Suburban
		Rural
	Hospital size	
	ICU bed to general floor bed ratio	
	Hospital category	
		Academic
		Community
		Hybrid
	Intermediary or observation units	
		Present
		Absent
	Intermediary care to ICU bed ratio	
ICU	ICU staffing models	
		High intensity
		Low intensity
	ICU bed occupancy rates	
	ICU type	Subspecialty specific
		Mixed
	ICU physician coverage model	“Closed” or intensivist primary coverage
		Co-management model
		“Open” coverage model
Patient	Average patient acuity	

*In general, factors can be divided into hospital specific, ICU specific and patient specific*.

A retrospective single center study (Mayo Clinic, Rochester, MN) characterized the percentage of LRM patients in their medical ICU as 22.1%, surgical ICU as 45.7%, mixed medical-surgical ICU as 30.7% with an overall average of 37.2% (17). ICUs specializing in postoperative care of complex surgical procedures like cardiothoracic procedures, transplantations and complex neurosurgical procedures in general and almost by definition have a low percentage of LRM patient stays, as their management will inevitably involve multiple active treatments addressing hemodynamic management or mechanical ventilation. Certain subspecialty ICUs are generally expected to host higher percentages of patient stays for purposes of maximal monitoring for deterioration/complication, i.e., monitoring for arrhythmias in cardiac care units, monitoring for changes in neurological status in Neuroscience ICUs or monitoring for limb or organ perfusion in surgical ICUs.

At WMC the Neuroscience Critical Care Unit consists of 17 ICU beds and 12 Intermediate Level Care beds (Step down beds). Only the 17 ICU beds are staffed by neurointensivists in an “open” co-management model, meaning Neurosurgery and Neurology patients are co-managed by Pulmonary/Critical Care consultants for the general Critical Care aspects of their care like ventilator management, best practices etc. The LRM to AT ratio of our Neuroscience ICU has to be kept in perspective to our ICU to stepdown bed ratio of 17:12 and the absence of a specialized stroke unit. LRM to AT ratios may be significantly different in care settings with different ICU to intermediate care level bed ratios or specialized stroke units.

Our institution started an electronic ICU program in January of 2016. The electronic ICU is staffed 24/7/365 by critical care trained nurses and doctors who monitor and assist the bedside ICU team without altering the bedside staffing model. The electronic ICU comprehensively evaluates all patients on admission to the ICU, monitors and assists with best practice compliance, standardizes care practices, monitors for any signs of decompensation and assists the bedside team in times of crisis. The electronic ICU also gathers and enters all data relevant for APACHE IVa risk predictive modeling (classification by surgical and non-surgical APACHE IVa admission diagnostic categories, comorbidities, admission source) and provides comprehensive quarterly performance reviews to ICUs (including observed and predicted ICU and hospital mortality, length of stay and ventilator durations, transfusion practices, glucose management, stress ulcer prophylaxis, venous thromboembolism prophylaxis, lung protective ventilation strategy).

56.9% of patients who were admitted to our Neuroscience ICU in 2016 and 2017 were classified as LRM, whereas 43.1% were classified as AT. A recently published single center large case series on 1,879 patients reports similar distributions of 46.5% LRM to 53.5% AT for a 16 bed academic hospital neurological ICU (18). The two most frequent admission diagnoses were the same for LRM and AT categories (stroke and intracranial hemorrhage/hematoma), illustrating that further differentiating factors like time from onset of symptoms, NIH stroke scale scoring and radiographic extent of brain tissue involvement determine whether a patient is a candidate for and will likely benefit from active treatment as opposed to monitoring only. Patients with intracranial hemorrhage were 1.4 times more likely to be actively treated than primarily monitored without ICU specific treatments (*p* = 0.017). Patients with SAH were represented in the respective surgical admission category for SAH if they were admitted to the Neuroscience ICU after surgical aneurysm clipping or neuroendovascular intervention for aneurysm/intracranial AVM. They were represented in the medical category for SAH if they did not undergo surgery or neuroendovascular intervention. Patients with surgically managed SAH were about twice as likely to be in the LRM group (postoperative observation) than in the AT group (postoperative active management) (5.6 vs. 2.9%, *p* = 0.008), whereas medically managed patients with SAH were about twice as likely to be in the AT group as compared to the LRM group (6.0 vs. 2.9%, *p* = 0.002). This inverse LRM-AT distribution between surgically and medically managed SAH patients may be attributable to more acute and initial presentations of SAH in the medical group requiring aggressive management vs. patients as opposed to more subacute patients in the surgical category who got admitted to the Neuroscience ICU after they had their aneurysm or AVM surgically secured, with their main focus being on prevention of complications. A plethora of postsurgical conditions and medical neurological conditions, jointly classified as “Postsurgical, other” or “Neurologic Medical, Other” were being admitted as LRM, which reflects the diversity of Neuroscience ICU patient populations in our tertiary and quaternary University hospital setting which do not fit more accurately in any of the other available 30 medical or 26 surgical APACHE IVa admission diagnosis categories (see Materials and Methods). Patients admitted to the Neuroscience ICU with Seizures were significantly more likely to be actively treated (4.9%) as compared to monitored (2.9%). The reason for this is that any pharmacologic treatment for seizures and metabolic encephalopathy counts as active treatment as per eCM criteria (see Table 1). This may have falsely lowered the LRM group in this particular ICU as compared to TISS standards. Patients in our Neuroscience ICU are co-managed by either Neurosurgery or Neurology Primary Attendings and Consulting Pulmonary/Critical Care/Neuro-Critical Care Certified Intensivists. There was a significantly disproportionate admixture of “boarding” patients primarily managed by Pulmonary & Critical Care attendings in the AT over the LRM group (5.2 vs. 2.1%; *p* = 0.0003), which likely caused an increase in actual and predicted ICU and hospital mortality, given that the medically critically ill patient population at our institution has high acuity scores and high mortality rates.

### Which practice guidelines and policy statements provide guidance for low risk monitoring in neuroscience ICUs?

There is clear evidence that patients with acute strokes have improved outcomes when treated in specialized stroke units or units with centralized expertise and case volume in stroke care (19, 20). The American Heart Association/American Stroke Association Guidelines for the early management of patients with acute ischemic stroke advocate admission to specialized ICU for monitoring of mental status and neurological deficits, as well as management of oxygenation levels, blood pressure, heart rate, body temperature and blood glucose levels as well as to monitor for complications from anticoagulation (21). It is well-known that after initial stroke assessment up to 26% of patients will have a subsequent deterioration in their status (10% due to progression of ischemic stroke, 10% due to cerebral edema and roughly 3% each due to secondary hemorrhagic or ischemic events) (21). The leading complications of acute ischemic strokes are related to dysphagia and/or alterations in mental status (pneumonitis, pneumonia), followed by heart failure and gastrointestinal bleeding (22). Interestingly, the guidelines do not directly address ICU level monitoring for patients specifically after systemic or local pharmacologic or mechanical thrombolysis, either, or intracranial acute angioplasty and stenting, but clinically it is intuitive that these post-procedural patients should be monitored for signs of side effects/complications. The complications of thrombolysis are symptomatic intracerebral hemorrhage, systemic hemorrhage events or thrombolytic related angioedema.

### Which acutely critically ill patient populations might be missed by the TISS/APACHE active treatment list and therefore misclassified as LRM?

The TISS list of active ICU treatments has last been updated in 1983. A plethora of new treatment modalities has entered the market since that time. A large number of new ICU treatments will, however, be indirectly captured as due to their invasive or complex nature they will need other treatments along with them that are captured by TISS, e.g., invasive neuro-monitoring will need mechanical ventilation, vasoactive infusions and sedation. TISS does not include frequent airway clearance/suctioning as an active treatment, which can often be problematic after neurologic injury. Given that this treatment modality usually applies to patients long after their first ICU day (after extubation or tracheostomy/ventilator weaning), misclassifications of patients who receive only this active treatment as LRM is extremely unlikely, given that the timeframe focus of the LRM metric is the first ICU day.

### How does LRM% relate to other ICU performance metrics?

A study by Zimmerman et. al. on 359,715 patients from 108 medical, surgical, and mixed medical-surgical ICUs has shown that the percentage of LRM patients is inversely correlated with ICU performance, i.e., the nine best performing units (lowest ICU and hospital mortality ratios and LOS) had the lowest LRM%, whereas the nine worst performing ICUs (highest ICU and hospital mortality and LOS ratios) had the highest LRM% for medical, surgical and mixed medical-surgical units alike (23).

### Strengths and limitations

The current study contains admission data for 2016–2017. This is an advantage as the admissions all represent the current care approach and effects of changes over time are minimal. The disadvantage lies in the fact that the overall admission numbers are lower than for studies over longer periods of time. Multiple year studies also can address the question of seasonal variations in LRM%. In addition, the aforementioned ICU in this citation is part of a 1,000 bed academic tertiary and quaternary care center with very high Case Mix Indices and may not be representative of different types of hospitals or hospital systems.

## Conclusions

Differentiating patients admitted to our Neuroscience ICU into those who received active ICU specific treatments during their first 24 h in the ICU (AT group, 43.1% of patients) and those who had a low ICU mortality risk (< 10%) *and* a primary focus on monitoring during their first 24 h in the ICU (LRM group, 56.9% of patients) was highly discriminatory for APACHE IVa acuity scores and all APACHE IVa derived risk predicted outcomes (actual and predicted ICU and hospital mortality). LRM Patients exhibited significantly lower APACHE IVa scores, ICU and hospital mortality rates when compared to actively treated patients. Observed-over-expected ICU and hospital mortality ratios were better than predicted by APACHE IVa for low risk monitored patients and close to prediction for actively treated patients. This constellation of findings suggests that at least a subset of our LRM patients may safely and more cost effectively be cared for in intermediate level care settings, enabling redistribution of ICU resources to higher acuity patients in need of ICU treatments. In addition, patients with Acute Strokes accounted for 391 out of 1,702 overall Neuroscience ICU admissions (23%) and were equally represented in both LRM (24.3%) and AT groups (21.3%) (*p* = 0.13). American Heart Association/American Stroke Association Guidelines advocate admission to specialized intensive care units or specialized stroke units with centralized expertise in the early management of acute strokes. Hospitals with high numbers of Acute Stroke patients like ours might benefit from the creation of specialized stroke units.

## Author contributions

CDB and CS conceptualized the study. CDB performed data extraction, review and statistical analyses. CDB prepared the manuscript draft. CDB, CB, CC, DC, MS, and CS provided revisions. All authors approved the final manuscript.

### Conflict of interest statement

The authors declare that the research was conducted in the absence of any commercial or financial relationships that could be construed as a potential conflict of interest.

## Abbreviations

ICU: intensive care unit
LRM: low risk monitor
AT: active treatment
APACHE: Acute Physiology, Age and Chronic Health Evaluation
SAH: subarachnoid hemorrhage
ICH: intracerebral hemorrhage
TISS: therapeutic intervention scoring system
LOS: length of stay
US: United States.
